# Incidence Trends of Early‐Onset Colorectal Cancer in Germany: A Registry‐Based Study From 2003 to 2023

**DOI:** 10.1002/ijc.70600

**Published:** 2026-06-23

**Authors:** Sven Voigtländer, Hiltraud Kajüter, Ina Wellmann, Andras Szentkirályi, Bernd Holleczek, Volker Arndt, Jacqueline Müller‐Nordhorn, the ALSTER Working Group, Josephine Kanbach, Sabine Luttmann, Meike Ressing, Cécile Ronckers, Soo‐Zin Kim‐Wanner, Alice Nennecke, Frederik Peters, Klaus Kraywinkel, Roxana Müller‐Eberstein, Hannah Baltus, Paula Grieger, Antje Schliemann, Annika Waldmann

**Affiliations:** ^1^ Bavarian Cancer Registry, Bavarian Health and Food Safety Authority Nuremberg Germany; ^2^ Cancer Registry of North Rhine Westphalia Bochum Germany; ^3^ Saarland Cancer Registry Saarbrücken Germany; ^4^ German Cancer Research Center (DKFZ), Epidemiological Cancer Registry Baden‐Württemberg Heidelberg Germany

**Keywords:** colorectal cancer, early‐onset, Germany, incidence, registries

## Abstract

Motivated by studies on early‐onset colorectal cancer (EO CRC, below 50 years) from the United States (US) and elsewhere, this study aimed to provide national incidence trends of EO CRC for Germany. We retrieved colorectal cancer cases (ICD‐10 codes C18‐C20) using pooled data from German cancer registries with high‐quality data. Appendix (C18.1) was excluded from the main analyses and investigated separately. We calculated age‐standardised incidence rates (ASR) by calendar year, age group, sex, tumour site, histological subtype, tumour size, grading and estimated corresponding average annual percent changes (AAPC). For comparison, US cancer registry data was drawn from the Surveillance, Epidemiology, and End Results programs 21 population‐based registries (SEER 21). From 2003 to 2023, ASRs of EO CRC (C18‐C20 excluding C18.1) increased for men (AAPC: 0.8%) and women (AAPC: 0.9%) in Germany. By 10‐year age groups, the increase was limited to men and women aged 20–29 years (AAPC males: 3.3%; AAPC females: 3.9%) and 30–39 years (AAPC males: 2.2%; AAPC females: 2.0%). For appendix cancer (C18.1), ASRs were rising for all age groups below 50 years of age. Increases for both colorectal cancer (excluding the appendix) and appendix cancer tended to be higher for cancers with good prognosis (neuroendocrine neoplasms, small size, low grading). In contrast to the US, Germany had a substantially lower EO CRC incidence in 2003 that increased at a slower pace. It is important to monitor future EO CRC incidence trends. The causes of the observed trends remain unclear.

## Abstract (highlights)

What's new?

The evidence on the rising incidence of colorectal cancer among adults below 50 years of age largely stems from the United States. Using high‐quality cancer registry data for the 2003–2023 period in Germany, this study found a moderate increase of early‐onset colorectal cancer incidence that was limited to those aged 20–39 years and more pronounced among the 20–29‐year‐olds. The increase, which was driven by adenocarcinomas and neuroendocrine neoplasms, was generally observed for cancers with good prognosis. The incidence of early‐onset colorectal cancer in Germany was substantially lower and increased at a slower pace than the US incidence.

## Graphical abstract

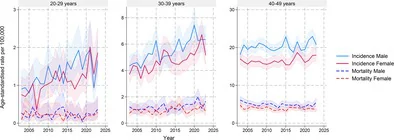

**Abbreviations**
95% CI: 95% confidence interval
AAPC: average annual percent change
APC: annual percent change
ASR: age‐standardised rate
BMI: body mass index
DCO: death certificate only
EO CRC: early‐onset colorectal cancer
FIT: faecal immunochemical test
FOBT: faecal occult blood test
ICD‐10: International Statistical Classification of Diseases and Related Health‐Problems, Tenth Revision
ICD‐O‐3: International Classification of Diseases for Oncology, Third Edition
NEC: neuroendocrine carcinoma
NEN: neuroendocrine neoplasm
NET: neuroendocrine tumour
SEER: US Surveillance, Epidemiology, and End Results program
WHO: World Health Organization
ZfKD: Centre for Cancer Registry Data (German: Zentrum für Krebsregisterdaten)

## 1 Introduction

Over the last years, a number of international studies have found increasing incidence rates of colorectal cancer among adults below 50 years of age, often described as early‐onset colorectal cancer (EO CRC), in both men and women [1, 2, 3, 4, 5, 6]. The relative increase in incidence has been higher for younger age groups, such as 20–29 years and 30–39 years [2, 3, 4]. Similar trends for young adults have been observed for Germany [7, 8]. Contrary to the increasing incidence rates among young adults, age‐standardised incidence rates of CRC over all ages have been declining for several decades in many countries, such as the United States [9], Canada, Australia [2] and Germany [10].

Tumour sites most affected by the increase of EO CRC differ between studies, which is partly due to differential data analysis [11]. Many studies, for instance, excluded or reported the appendix separately because appendix cancer has been affected by classification changes and differs from CRC in terms of aetiology and biological behaviour [2, 4, 7, 8, 12, 13, 14, 15]. Most studies that excluded the appendix reported a more pronounced increase for rectal cancer compared with colon cancer [11]. For the US Surveillance, Epidemiology, and End Results program (SEER), for instance, the annual percent changes (APC) of rectal cancer incidence were 3.2% for the age group 20–29 years (period 1974–2013) and 3.2% for the age group 30–39 years between (period 1980–2013), while the corresponding APCs for colon cancer were 2.4% (period 1983–2013) and 1.0% (period 1988–2013) [4]. Studies on appendix cancer incidence trends alone showed large relative increases starting in the 1990s and exceeding those of rectal and colon cancer [12, 14]. These increases were not limited to young adults [14].

Histological subtype of EO CRC cases is predominantly adenocarcinoma [13, 16] except for the appendix [8], where neuroendocrine neoplasms (NEN) are more common. NEN can be grouped into neuroendocrine tumours (NETs) and neuroendocrine carcinoma (NEC), whereby NETs show a substantially better prognosis compared with adenocarcinomas as well as NECs [13]. However, histological subtype stratification regarding trends of EO CRC is scarce. Three studies, one from SEER [16] and two from the most populous German states [8, 13], showed that the proportion of NEN among CRC increased in all age groups while the proportion of adenocarcinomas decreased. This trend was more pronounced for rectum compared to colon (without appendix) [11, 16]. For this reason, it is necessary to account for histological subtype in EO CRC studies to avoid confounding by histology [17].

Causes of the increasing incidence of EO CRC are not adequately understood. Most cases occur sporadically and up to 30% have an inherited component, that is, a hereditary cancer syndrome or a family history of CRC [6]. Apart from genetic and familial risk factors, the EO CRC patient population comprises patients at increased CRC risk due to known risk factors such as inflammatory bowel disease as well as patients without a predisposition [1]. The observed birth cohort effect in combination with a long latency period points to major lifestyle changes beginning in the 1950s like childhood and adolescent obesity, antibiotic use, low physical activity, unhealthy diet and reproductive technology potentially leading to early‐life physiological or metabolic changes affecting the gut microbiome [6, 18, 19]. Recent studies have found *Escherichia coli* in the gut microbiota that carry the polyketide synthase (pks) island, which leads to the production of Colibactin and drives mutational processes [20]. Diagnosis of EO CRC tends to occur due to the onset of signs and symptoms in a later cancer stage compared with average‐onset CRC [6].

CRC Screening in Germany currently starts at the age of 50 including faecal occult blood test (FOBT, since 1977, every 2 years) and colonoscopy (since 2002, two every 10 years) [21, 22]. Recently, there have been a number of changes such as the replacement of FOBT by faecal immunochemical test (FIT) in 2017, the reduction of the colonoscopy start age from 55 to 50 years for men in 2019 and for women in 2025, as well as the implementation of an invitation procedure in 2019. For risk groups with a CRC predisposition, such as polyposis, colitis ulcerosa, or a family history of CRC, German guidelines recommend colonoscopy by the age of 40 at the latest [23].

Most detailed EO CRC analyses and a large part of the evidence so far stem from the United States, while similarly detailed studies are scarce for European countries and Germany as part of it. For instance, existing studies on EO CRC in Germany are characterised by various limitations, such as the analysis of a single state [8, 13], no differentiation between colorectum and appendix [7], the neglect of histological subtypes [7] or the focus on a single subtype [24] as well as a wide range of age groups that were analysed [13]. Thus, our study aimed to provide national trends of CRC incidence and mortality for Germany up to the year 2023, focusing on age groups below 50 years of age according to tumour site and histological subtype. This may help to understand the nature of EO CRC trends in Germany and the factors driving those trends. We also aimed to compare overall EO CRC incidence trends in Germany with the United States, where the increase of EO CRC was reported first [25].

## 2 Material and Methods

### 2.1 Data

We retrieved national data on incident CRC (codes C18‐C20 of the International Statistical Classification of Diseases and Related Health‐Problems, Tenth Revision, ICD‐10) from the Centre for Cancer Registry Data (Zentrum für Krebsregisterdaten [ZfKD]) at the Robert Koch Institute as of 20 June 2025 [26]. We did not restrict the data regarding age, death certificate only (DCO) cases or multiple primary cancers. The ZfKD data is based on data from population‐based cancer registries at the federal state level, which were initially established by state initiative beginning in the 20th century and have been mandatory since 2009 for all states based on national law [27]. The selection of the study period and states was based on quality indicators, that is, completeness of coverage and proportion of DCO cases [27], with the aim to attain a large study population with a preferably long study period. Based on these criteria we included the states Bavaria, Brandenburg, Bremen, Hamburg, Lower Saxony, Mecklenburg‐Vorpommern, Saarland, Saxony and Schleswig‐Holstein, with a completeness of 90% or more and a proportion of DCO cases of less than 15% (among those aged 20–49 years) for each year of the period 2003–2023. For the largest state North Rhine‐Westphalia, which reached completeness of coverage in 2008, we included the administrative district of Münster that fulfilled the criteria in 2003 and beyond. Hereafter, we use the term state to refer to German federal states and the administrative district of Münster. The analysis set covered 46% of the German population of 83.3 million people as of 2023. Although the inclusion of North Rhine‐Westphalia would have increased the covered population, given that it represents 21.6% of the German population, doing so would have shortened the study period by 5 years. The population data based on the latest census in 2022 as well as the colorectal cancer mortality data for the study period and study states were also retrieved from the ZfKD.

For the comparison of EO CRC incidence trends in Germany and the United States, we additionally retrieved US data from SEER*Explorer for incidence based on SEER 21 and for mortality based on US mortality [28]. SEER 21 is the largest SEER database covering 21 US population‐based cancer registries with incidence data available from 2000 onwards (https://seer.cancer.gov/registries/terms.html).

### 2.2 Variables

We stratified by years of age at diagnosis (20–29, 30–39, 40–49 years, 50 years and above) and sex (male, female). Tumour site of CRC was grouped into proximal colon (ICD‐10 codes C18.0, C18.2‐C18.4), distal colon (C18.5‐C18.7), overlapping lesion of colon (C18.8), not otherwise specified colon (C18.9) and rectum (C19, C20). Appendix (C18.1) was considered separately. Categorisation of histological subtype was based on the International Classification of Diseases for Oncology, Third Edition (ICD‐O‐3) [29], into adenocarcinomas (histological codes: 8140‐8149, 8160‐8163, 8190‐8221, 8250‐8552, 8570‐8576, 8940‐8941, 9110) and NEN (8013, 8041, 8152, 8154, 8240, 8241, 8244, 8245, 8246, 8249). The remaining histological codes were grouped into unspecified cancers (8000‐8005, 8010‐8012, 8014‐8015, 8020‐8022, 8050) and other specific cancers (all other histological codes). For the appendix, cases with the histological code 8240 that had been classified as ‘borderline malignant’ (behaviour code 1) in earlier years of this study were included due to a respective behaviour code change to ‘malignant’ (behaviour code 3) from ICD‐O‐3 to its first revision in 2013 [29]. Moreover, we stratified for tumour size (T category: T1, T2, T3, T4, Unknown) and grading (Low: G1, Intermediate: G2, High: G3/G4, Unknown) according to the TNM Classification of Malignant Tumours (8th edition) [30].

For comparison of Germany with the United States, we used colorectal cancer cases (ICD codes C18‐C20 excluding C18.1) below 50 years of age at diagnosis and stratified by sex (male, female), which was available in both databases. Cases were not grouped according to histological subtype, tumour size or grading.

### 2.3 Statistical Analysis

We calculated age‐standardised rates (ASRs) for incidence and mortality per 100,000 person‐years based on the European standard population [31] with 95% confidence intervals (95% CI) [32] by calendar year, age group, sex, and, with regard to incidence, additionally by tumour site, histological subtype, tumour size and grading. DCO cases were included in the analysis of incidence to avoid underestimation. To analyse long‐term trends, we calculated average annual percent changes (AAPCs) with 95% CIs [33]. AAPCs are a weighted average of annual percent changes (APCs) from a Joinpoint model, also known as segmented regression, which is a log‐linear regression of ASRs with year of diagnosis as independent variable. We performed separate analyses for the colorectum (ICD‐10 codes C18‐C20 excluding C18.1) and the appendix (ICD‐10 code C18.1). As cause of death data was only available in terms of three digits ICD‐10 codes, we could only analyse mortality of tumours of the entire colorectum including the appendix.

For comparability of EO CRC incidence trends between Germany and the United States, we calculated ASRs using the 2000 US standard population [34] as SEER*Explorer provided only ASRs accordingly based on the SEER 21 registries from 2000 to 2022 [28]. Trend analysis was based on APCs excluding 2020 incidence rates in order to have estimates similar to the trend data published by SEER*Explorer. Comparison was limited to the colorectum (ICD‐10 codes C18‐C20 excluding C18.1) as separate data for the appendix was not available. Additionally, we similarly compared EO CRC mortality trends between Germany and the United States from 2000 to 2023. As SEER*Explorer only published US mortality and no mortality estimates restricted to the SEER 21 registries, we also used nationwide German mortality data (including all federal states) for consistency.

ASR analyses were performed using Stata v19.0 (https://www.stata.com/) and AAPC analyses using Joinpoint Regression Programme v5.4 from the US National Cancer Institute [35].

## 3 Results

### 3.1 Study Population

We identified 28,046 colorectal cancers in 27,568 persons aged 20–49 years within the study period 2003–2023. Most cases were observed in the age group 40–49 years (*n* = 21,653; 77.2%) and among males (*n* = 15,621; 55.7%) (Table 1). 12,438 cases (44.3%) were localised in the rectum, followed by 7766 cases (27.7%) in the distal colon and 6177 cases (22.0%) in the proximal colon. The proportion of rectal cancers was higher in men, while the proportion of distal colon cancers was higher in women. In terms of histological subtype, 25,327 cases (90.3%) were adenocarcinomas, 1351 cases (4.8%) NEN, 231 cases (0.8%) other specific cancers and 1137 cases (4.1%) unspecified cancers. The proportion of adenocarcinoma increased, while the proportion of NEN decreased with age regarding all histological subtypes. The most frequent tumour size was T3 (*n* = 12,137; 43.3%) followed by unknown T category (*n* = 5321; 19.0%). The most frequent grading was intermediate (*n* = 16,005; 57.1%), though the proportion of low and high grading was higher in the younger age groups. Within the subgroup of colorectal NEN, a different distribution with a higher proportion of rectal cancers (74.9%), T1 cancers (37.3%), cancers with unknown T category (44.8%) and cancers with low grading (62.4%) was observed (data not shown).

**TABLE 1:** Characteristics of incident colorectal cancer for ages 20–49 in Germany, 2003–2023.

	20–29 years				30–39 years				40–49 years				Total			
	Male		Female		Male		Female		Male		Female		Male		Female	
	*n*	%	*n*	%	*n*	%	*n*	%	*n*	%	*n*	%	*n*	%	*n*	%
Total	633	100	499	100	2882	100	2379	100	12,106	100	9547	100	15,621	100	12,425	100
Tumour site																
Proximal colon	161	25.4	127	25.4	745	25.9	531	22.3	2587	21.4	2026	21.2	3493	22.4	2684	21.6
Distal colon	156	24.6	160	32.1	701	24.3	715	30.1	3102	25.6	2932	30.7	3959	25.3	3807	30.6
Rectum	265	41.9	181	36.3	1242	43.1	952	40.0	5765	47.6	4033	42.2	7272	46.6	5166	41.6
Overlapping lesion	12	1.9	6	1.2	30	1.0	27	1.1	90	0.7	81	0.8	132	0.8	114	0.9
Unspecified	39	6.2	25	5.0	164	5.7	154	6.5	562	4.6	475	5.0	765	4.9	654	5.3
Histological subtype																
Adenocarcinoma	515	81.4	405	81.2	2558	88.8	2077	87.3	11,069	91.4	8703	91.2	14,142	90.5	11,185	90.0
Neuroendocrine neoplasm	78	12.3	56	11.2	186	6.5	176	7.4	446	3.7	409	4.3	710	4.5	641	5.2
Other specific	4	0.6	7	1.4	17	0.6	29	1.2	94	0.8	80	0.8	115	0.7	116	0.9
Unspecified	36	5.7	31	6.2	121	4.2	97	4.1	497	4.1	355	3.7	654	4.2	483	3.9
Tumour size																
T1	78	12.3	64	12.8	260	9.0	295	12.4	1332	11.0	1071	11.2	1670	10.7	1430	11.5
T2	52	8.2	43	8.6	253	8.8	242	10.2	1184	9.8	1057	11.1	1489	9.5	1342	10.8
T3	255	40.3	189	37.9	1284	44.6	961	40.4	5312	43.9	4136	43.3	6851	43.9	5286	42.5
T4	118	18.6	86	17.2	489	17.0	393	16.5	1982	16.4	1589	16.6	2589	16.6	2068	16.6
Unknown	130	20.5	117	23.4	596	20.7	488	20.5	2296	19.0	1694	17.7	3022	19.3	2299	18.5
Grading																
Low	127	20.1	81	16.2	394	13.7	324	13.6	1192	9.8	991	10.4	1713	11.0	1396	11.2
Intermediate	258	40.8	232	46.5	1407	48.8	1272	53.5	7144	59.0	5692	59.6	8809	56.4	7196	57.9
High	167	26.4	110	22.0	744	25.8	525	22.1	2514	20.8	1961	20.5	3425	21.9	2596	20.9
Unknown	81	12.8	76	15.2	337	11.7	258	10.8	1256	10.4	903	9.5	1674	10.7	1237	10.0

*Note:* T, tumour size according to the TNM Classification of Malignant Tumours (8th edition). ICD‐10 codes C18‐C20 excluding C18.1.

The frequency distributions for appendix cancers are shown in Table S1. We identified 2969 cases in total. In contrast to colorectal cancers, appendix cancers were more equally distributed across the 10‐year age groups, more frequent among women compared with men and most cases were NEN (*n* = 2071; 69.8%), though their proportion was decreasing with age. We also observed more T1 cancers as well as cancers with low grading compared with colorectal cancers.

### 3.2 Age‐Standardised Rates and Annual Percent Change of Incidence and Mortality by Age Group and Sex

**FIGURE 1:**
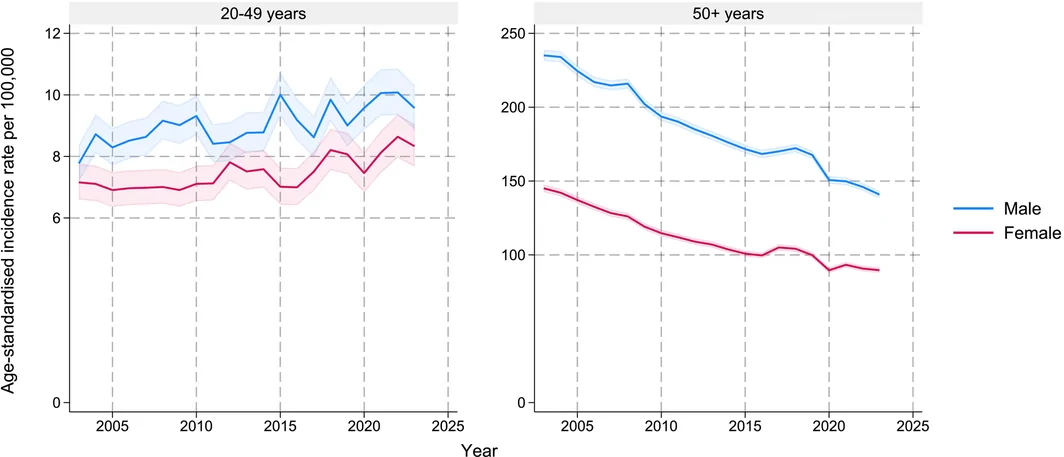
Age‐standardised incidence rates with 95% confidence intervals of colorectal cancer for ages 20–49 and 50+ in Germany, 2003–2023, by sex. ICD‐10 codes C18‐C20 excluding C18.1. Age‐standardised incidence rates per 100,000 based on the European standard population (1976). Scale of *y*‐axis varies.

Between 2003 and 2023, ASRs of colorectal cancer incidence increased for ages 20–49 years for both males (2003: 7.8 per 100,000 [95% CI: 7.2 to 8.4]; 2023: 9.6 per 100,000 [95% CI: 8.9 to 10.3]) and females (2003: 7.2 per 100,000 [95% CI: 6.6 to 7.8]; 2023: 8.3 per 100,000 [95% CI: 7.7 to 9.0]) (Figure 1). The increase was similar for men (AAPC: 0.8%; 95% CI: 0.3 to 1.3) and for women (AAPC: 0.9% [95% CI: 0.5 to 1.3]) (Table 2). In contrast, corresponding ASRs for those aged 50 years and above substantially declined for males (AAPC: −2.7% [95% CI: −2.9 to −2.5]) and females (AAPC: −2.4% [95% CI: −2.6 to −2.1]) (data not shown).

**FIGURE 2:**
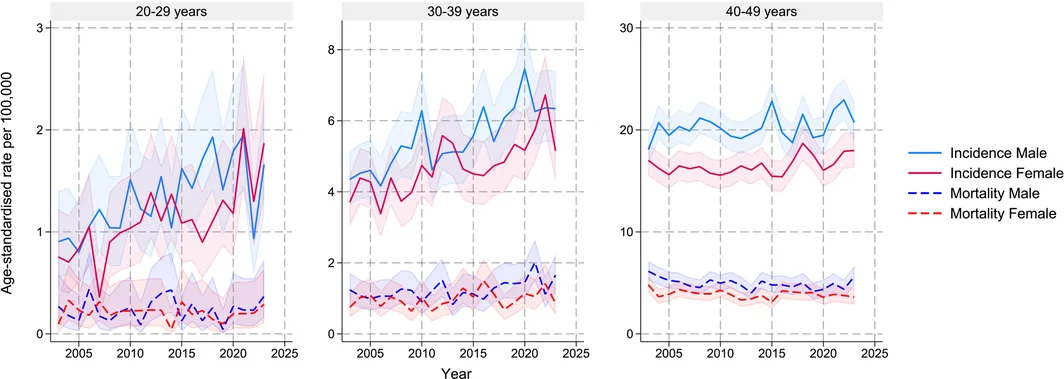
Age‐standardised incidence and mortality rates with 95% confidence intervals of colorectal cancer for ages 20–49 in Germany, 2003–2023, by sex. Incidence for ICD‐10 codes C18‐C20 excluding C18.1 and mortality for ICD‐10 codes C18‐C20 including C18.1. Age‐standardised incidence and mortality rates per 100,000 based on the European standard population (1976). Scale of *y*‐axis varies.

Figure 2 shows the ASRs of EO CRC incidence and mortality by 10‐year age groups. Incidence per 100,000 was highest in the age group 40–49 years. The relative ASR increase, however, was highest for those aged 20–29 years (AAPC males: 3.3% [95% CI: 1.6 to 5.1]; AAPC females: 3.9% [95% CI: 2.3 to 5.7]) followed by those aged 30–39 years (AAPC males: 2.2% [95% CI: 1.5 to 2.9]; AAPC females: 2.0% [95% CI: 1.0 to 3.1]) (see also Table S2). For the age group 40–49 years incidence remained stable (AAPC males: 0.4% [95% CI: −0.3 to 1.0]; AAPC females: 0.4% [95% CI: −0.0 to 0.8]), though the incidence from 2017 onwards tended to be slightly higher than before. ASRs of EO CRC incidence were approximately four times higher than the ASRs of EO CRC mortality. There was no clear mortality trend for the age group 20–29 years, while mortality increased for males aged 30–39 years (AAPC: 2.0% [95% CI: 0.7 to 3.4]) but not females (AAPC: 1.3% [95% CI: −0.6 to 3.2]) and declined for males aged 40–49 years (AAPC: −1.1% [95% CI: −1.9 to −0.2]) but not females (AAPC: −0.6% [95% CI: −1.6 to 0.3]).

For appendix cancers, the ASRs of incidence and their trend were rather similar for the 10‐year age groups between 20 and 49 years (Table S3, Figure S1). The ASRs were below 1.0 per 100,000 in 2003 and increased to about 1.5 per 100,000 until the years 2012–2014 and remained stable to the end of the study period.

### 3.3 Incidence Trends by Tumour Characteristics

**FIGURE 3:**
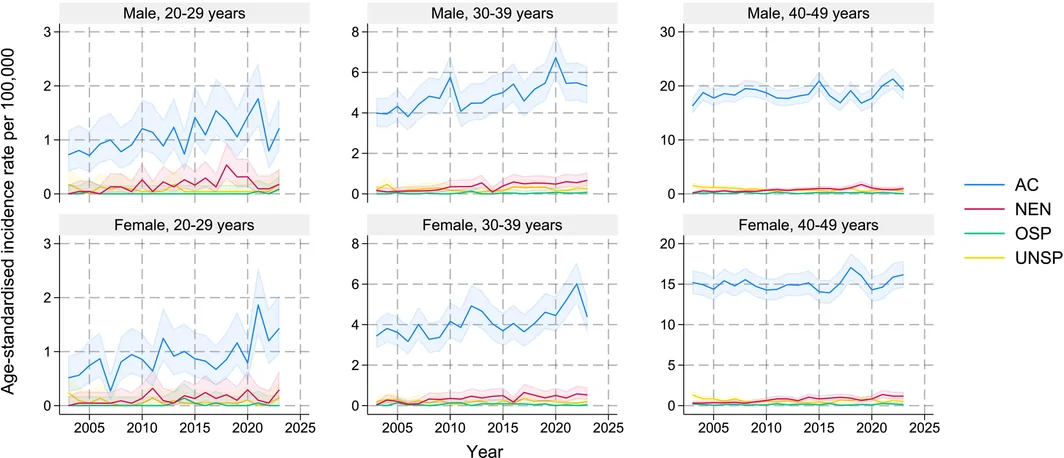
Age‐standardised incidence rates with 95% confidence intervals of colorectal cancer for ages 20–49 in Germany, 2003–2023, by sex and histological subtype. ICD‐10 codes C18‐C20 excluding C18.1. AC, adenocarcinomas; NEN, neuroendocrine neoplasms; OSP, other specific cancers; UNSP, unspecified cancers. Age‐standardised incidence rates per 100,000 based on the European standard population (1976). Scale of *y*‐axis varies.

Stratified by tumour site, AAPCs for rectum cancer were higher for women than for men in the age group 20–29 years. AAPCs for men and women aged 30–39 years were lower for proximal colon compared with distal colon and rectum (Table 2). For the age group 40–49 years, we observed a slight increase for proximal and distal colon in men and a slight increase for rectum cancers in women. Incidence of cancers with unspecified tumour site tended to decrease across all age groups (see also Figure S2). Figure 3 shows the ASRs of EO CRC incidence according to histological subtype. AAPCs for adenocarcinomas were similar to the overall AAPCs while those for NEN were much higher for the age groups 30–39 and 40–49 years. For the age group 20–29, no AAPCs were estimated for NEN due to years with zero cases. As for histological subtype, ASRs of unspecified histological subtype declined over the study period for females aged 30–39 years as well as males and females aged 40–49 years. Regarding tumour size, we observed a substantial increase of ASR for T1 independent of age, while the AAPCs were stable for T2 as well as T3 and positive for T4 in males and females aged 30–39 years (Table 2, Figure S3). A similar trend was observed for grading with low grading cancers having AAPCs well above‐average, intermediate grading cancers slightly being below‐average and high grading cancers having negative AAPCs for the age groups 30–39 and especially 40–49 (Table 2, Figure S4). Half of AAPCs for tumour size and grading could not be estimated for the age group 20–29 due to years with zero cases.

**TABLE 2:** Average annual percentage change with 95% confidence intervals for age‐standardised incidence and mortality rates of colorectal cancer for ages 20–49 in Germany, 2003–2023.

Incidence	20–29 years				30–39 years				40–49 years				Total			
	Male		Female		Male		Female		Male		Female		Male		Female	
	AAPC	95% CI	AAPC	95% CI	AAPC	95% CI	AAPC	95% CI	AAPC	95% CI	AAPC	95% CI	AAPC	95% CI	AAPC	95% CI
Total	3.3	[1.6; 5.1]	3.9	[2.3; 5.7]	2.2	[1.5; 2.9]	2.0	[1.0; 3.1]	0.4	[−0.3; 1.0]	0.4	[−0.0; 0.8]	0.8	[0.3; 1.3]	0.9	[0.5; 1.3]
Tumour site																
Proximal colon	2.8	[0.3; 5.6]	4.8	[1.1; 9.5]	1.3	[−0.1; 2.8]	1.3	[−0.4; 3.0]	0.9	[0.1; 1.7]	0.3	[−0.8; 1.4]	1.1	[0.3; 1.8]	0.8	[0.1; 1.4]
Distal colon	3.2	[1.0; 6.1]	3.9	[1.7; 6.7]	2.9	[1.4; 4.6]	2.8	[1.4; 4.4]	1.2	[0.2; 2.2]	0.5	[−0.4; 1.3]	1.7	[1.1; 2.3]	1.2	[0.6; 1.9]
Rectum	1.5	[−2.7; 10.6]	8.0	[6.0; 11.4]	2.6	[0.9; 4.5]	2.8	[1.4; 4.3]	−0.0	[−0.6; 0.5]	0.9	[0.3; 1.4]	0.6	[−0.0; 1.3]	1.4	[0.9; 1.9]
Overlapping lesion	NA		NA		NA		NA		0.1	[−4.5; 4.2]	NA		0.2	[−3.1; 3.4]	3.4	[−0.7; 8.4]
Unspecified	NA		NA		−1.8	[−5.0; 1.0]	−3.4	[−7.8; −0.0]	−3.0	[−5.1; −1.2]	−4.4	[−6.7; −2.6]	−2.7	[−4.3; −1.4]	−3.9	[−6.2; −2.1]
Histological subtype																
Adenocarcinoma	3.0	[1.2; 5.0]	4.0	[2.0; 6.4]	1.8	[1.0; 2.6]	1.9	[0.6; 3.1]	0.3	[−0.3; 0.9]	0.2	[−0.3; 0.7]	0.7	[0.1; 1.3]	0.7	[0.4; 1.1]
Neuroendocrine neoplasm	NA		NA		9.1	[6.1; 14.7]	4.7	[2.5; 7.8]	4.1	[1.1; 8.1]	7.7	[5.6; 10.9]	5.4	[2.6; 8.7]	8.0	[5.6; 12.6]
Other specific	NA		NA		NA		NA		1.3	[−4.3; 7.0]	1.3	[−2.8; 5.5]	1.1	[−4.0; 6.6]	1.5	[−2.2; 5.3]
Unspecified	NA		NA		−0.3	[−4.1; 3.7]	−1.0	[−4.4; 2.1]	−3.4	[−5.4; −1.4]	−2.8	[−5.4; −0.1]	−2.8	[−4.6; −1.1]	−2.6	[−4.7; −0.3]
Tumour size																
T1	NA		NA		6.0	[4.4; 8.2]	6.1	[2.6; 11.6]	3.8	[0.8; 6.5]	2.5	[1.3; 3.8]	4.8	[3.6; 6.7]	3.4	[2.5; 4.4]
T2	NA		NA		0.2	[−2.1; 2.3]	0.9	[−1.8; 3.6]	−1.1	[−2.3; 0.3]	−0.6	[−2.3; 1.0]	−0.3	[−2.1; 1.4]	0.0	[−1.5; 1.4]
T3	2.2	[0.4; 4.2]	1.5	[−1.5; 4.6]	1.5	[0.2; 2.9]	1.3	[−0.2; 2.7]	−0.0	[−0.8; 0.7]	−0.4	[−1.0; 0.1]	0.4	[−0.2; 0.9]	0.1	[−0.5; 0.6]
T4	2.5	[−1.7; 7.5]	NA		2.2	[0.2; 4.5]	3.1	[0.2; 6.3]	0.9	[−0.5; 2.2]	0.9	[−0.0; 1.8]	1.3	[0.6; 2.0]	1.5	[0.5; 2.5]
Unknown	1.7	[−2.8; 6.7]	4.4	[0.9; 7.5]	2.9	[1.6; 4.2]	1.4	[−0.3; 3.3]	0.6	[−0.6; 1.5]	1.8	[0.5; 2.8]	1.1	[0.0; 1.9]	2.1	[0.7; 3.0]
Grading																
Low	NA		NA		9.5	[7.8; 12.1]	7.3	[5.3; 10.1]	7.5	[6.6; 8.6]	6.9	[5.6; 8.5]	8.2	[6.9; 9.7]	7.4	[6.4; 8.7]
Intermediate	2.0	[−1.7; 6.3]	2.6	[0.1; 5.4]	1.4	[0.2; 2.6]	1.5	[−0.0; 3.0]	−0.2	[−0.8; 0.3]	0.2	[−0.3; 0.6]	0.2	[−0.3; 0.6]	0.5	[0.1; 1.0]
High	−3.1	[−10.0; 6.3]	2.9	[−1.3; 7.4]	−0.9	[−2.4; 0.5]	−0.5	[−2.5; 1.5]	−0.9	[−2.6; 0.4]	−2.5	[−3.6; −1.7]	−0.8	[−3.2; −1.0]	−1.9	[−2.9; −0.9]
Unknown	NA		NA		3.6	[1.9; 5.6]	3.4	[1.5; 5.5]	0.4	[−1.5; 2.4]	0.2	[−1.9; 2.8]	0.4	[−0.9; 1.7]	0.6	[−0.9; 2.1]

Mortality	20–29 years				30–39 years				40–49 years				Total			
	Male		Female		Male		Female		Male		Female		Male		Female	
	AAPC	95% CI	AAPC	95% CI	AAPC	95% CI	AAPC	95% CI	AAPC	95% CI	AAPC	95% CI	AAPC	95% CI	AAPC	95% CI
Total^a^	0.5	[−3.4; 4.7]	−0.7	[−2.9; 1.4]	2.0	[0.7; 3.4]	1.3	[−0.6; 3.2]	−1.1	[−1.9; −0.2]	−0.6	[−1.6; 0.3]	−0.2	[−1.1; 0.7]	−0.2	[−1.0; 0.5]

*Note:* T, tumour size according to the TNM Classification of Malignant Tumours (8th edition); NA, not applicable due to years with zero cases. ICD‐10 codes C18‐C20 excluding C18.1. Age‐standardised rates per 100,000 based on the European standard population (1976).

Abbreviations: AAPC, average annual percent change; CI, confidence interval.

^a^ Mortality due to ICD‐10 codes C18‐C20 including C18.1.

Regarding appendix cancers, only a few AAPCs could be estimated due to many categories that had years with zero cases (Table S4). AAPCs for adenocarcinoma as well as NEN were very similar to the overall AAPCs. For unknown T category as well as unknown grading, we found negative AAPCs for males and females aged 30–39 years and females aged 40–49 years, while AAPCs for low grading were positive across all age groups and tended to be higher than overall AAPCs.

### 3.4 Comparison of EO CRC Incidence and Mortality Trends Between Germany and the United States

**FIGURE 4:**
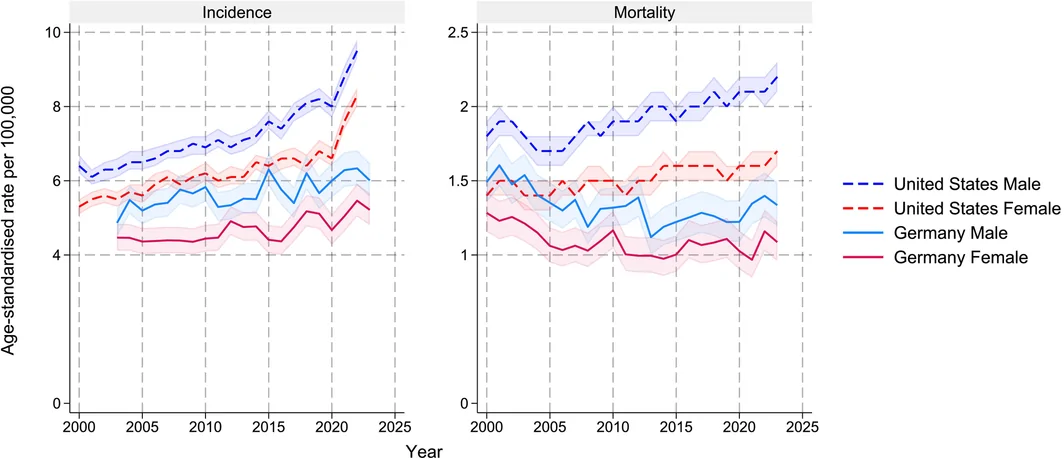
Age‐standardised incidence and mortality rates with 95% confidence intervals of colorectal cancer below age 50 in Germany and the United States, 2000–2023, by sex. Incidence for ICD‐10 codes C18‐C20 excluding C18.1 and mortality for ICD‐10 codes C18‐C20 including C18.1. Age‐standardised incidence rates per 100,000 based on the 2000 US standard population. Germany: Incidence based on analysis set and mortality based on all states; United States: Incidence based on SEER 21 registries and mortality based on all states. Scale of *y*‐axis varies.

Figure 4 shows the sex‐stratified ASRs of incidence and mortality for those below 50 years of age in Germany and the United States. Both the ASRs of incidence and their slope were lower in Germany than in the United States for both men and women (Table S5). In addition, there was an increase in trend for men in the year 2016 and women in the year 2019 in the United States but not in Germany, where we identified no Joinpoints. Regarding mortality, ASRs for men and women were also lower in Germany than in the United States, and the difference tended to increase over time (Figure 4). In men, we observed Joinpoints followed by an increase from 2014 in Germany and from 2004 in the United States, while there was no increase in Germany and a slight increase in the United States for mortality in women (Table S6).

## 4 Discussion

This study observed a moderate increase of EO CRC incidence in Germany, which was, based on the stratified analysis by 10‐year age groups, limited to those aged 20–39 years with a stronger rise in the age group 20–29 years. The increase was driven by adenocarcinomas as well as NENs and it was generally observed for cancers with good prognosis, that is, NENs, cancers of small tumour size and cancers with low grading. Though cancers with good prognosis were also found to increase in the age group 40–49 years, the overall incidence did not increase within this age group due to a parallel decrease in the incidence of cancers with high grading, unspecified site as well as unspecified histological subtype. EO CRC mortality in Germany did not change (age group 20–29 years), increased slightly (30–39 years) or declined slightly (40–49 years) so that, consequently, the ratio of mortality to incidence declined over the study period. In contrast to Germany, EO CRC incidence in the United States was already substantially higher in the past and increased at a faster pace over the study period. Regarding appendix cancer, incidence was rising for all age groups up to 50 years of age, though the rise was predominantly observed in the years 2003–2012.

In addition to confirm previous findings from Germany [7, 8, 13, 24], our results also cover recent years that include the introduction of the German CRC Screening programme. We also added EO CRC estimates for both colorectal and appendix cancer stratified by histological subtype. An earlier study using German cancer registry data showed that the increase in colorectal NENs is primarily due to an increase in well‐differentiated NETs that have a substantially better prognosis than adenocarcinoma and NECs [36]. Similar to our findings, previous studies from Germany [7, 24] and other Western countries [2, 3, 4] reported largest relative incidence increases for the youngest age groups with increases diminishing in older age groups. In contrast to countries, such as the United States, New Zealand or Canada [2, 4], however, studies from Germany [7, 24], including our results, have shown a stable CRC incidence among those aged 40–49 years (after control of confounding by appendix and NEN). Regarding tumour site, we did not observe a distinctly higher increase of colon cancer compared with rectal cancer in the age groups 20–29 and 30–39 years. This finding is in line with most previous studies [2, 4, 8, 12, 24] and contradicts other studies [3] that were confounded by appendix.

Several hypotheses have been raised to explain the increase of EO CRC ranging from hereditary factors and inflammatory bowel disease to lifestyle factors affecting early‐life physiological processes [1, 6, 19] as well as potential artefacts like confounding by histology [17] and earlier detection. Obesity, which is linked to other lifestyle factors such as low physical activity and unhealthy diet, represents a state of chronic inflammation [6] and is regarded as the most likely hypothesis for EO CRC incidence increase [19]. Studies have shown that obesity, specifically at a young age, increases the risk of EO CRC [37, 38]. In Germany, obesity prevalence has increased in the age group 25–34 years for males and females between 1990/1992 and 2008/2011 from 11% to 17% and 9% to 14% based on a body mass index (BMI) of 30 or more, respectively [39], but has been stable at around 6% for children aged 3–17 years between 2003/2006 and 2014/2017 based on a BMI at or above the 97th percentile of sex‐specific BMI‐for‐age [40]. In the United States, obesity prevalence increased in the age group 20–39 years between 2007/2008 and 2015/2016 from 31% to 36% based on a BMI of 30 or more and for those aged 2–19 years from 17% to 19% based on a BMI at or above the 95th percentile of sex‐specific BMI‐for‐age [41]. Though the numbers are not exactly comparable, obesity prevalence in Germany was much lower than in the United States prior to and during the observed period of time and it might have contributed to the substantially lower EO CRC incidence in Germany. In addition, EO CRC mortality in the United States increased on average by 1.2% per year between 2004 and 2020 [9], which was not confirmed by our results for Germany.

Earlier detection seems to have influenced our findings, given the increase in incidence of cancers with good prognosis in all 10‐year age groups below 50 years. Improved early detection is also plausible in view of the nearly stable mortality that did not parallel the increasing incidence. Assessing the role of the CRC screening programme regarding EO CRC incidence trends in Germany, however, is challenging as the program starts at age 50 and there is a lack of data on opportunistic screening and related time trends. German claims data analyses showed that 10‐year prevalence of colonoscopy was around 10% for the age groups 30–34 and 35–39 years and around 15% for the age groups 40–44 and 45–49 years [42, 43], which is quite substantial. The use of FOBT or FIT seemed to play no role [43]. Recent changes of the CRC screening programme in Germany, that is, the reduction of the colonoscopy start age for men from 55 to 50 years in 2019 and the implementation of an invitation procedure in same year may also have increased the use of FIT and colonoscopy in younger age groups, especially those aged 45–49 years. The slight increase in the sex‐specific incidence trends for the age group 40–49 years from 2017 might be an indication for this phenomenon. The interruption in the trend in 2020 is probably due to the COVID‐19 pandemic, which impaired cancer diagnosis [44]. Another factor boosting earlier detection of CRC cases among young adults may have been the broadly based long‐time awareness campaigns by the Burda Foundation that deliberately addressed people below 50 years and may have increased their likelihood of seeking medical care in case of symptoms or family members being diagnosed with CRC [24]. In the United States, the recent EO CRC incidence increase following 2020, the first year of the COVID‐19 pandemic, is likely due to a new recommendation by the US Preventive Services Task Force to include adults aged 45–49 years in CRC screening [45].

Lowering the start age for CRC screening to 45 years is not supported by our study as EO CRC incidence in the age group 40–49 years has been stable for the last 20 years and, more importantly, as the net benefit of screening individuals below 50 years with no suspicion of CRC and no specifically increased CRC risk in Germany is currently unclear [45, 46]. Furthermore, other preconditions for implementing or changing a screening programme [47, 48] are not met in several respects. First, Germany is far from having implemented all practicable, cost‐effective preventive strategies, which is evidenced by a recent comparison of prevention policy among 18 European countries including physical activity and diet that ranks Germany 17th reaching 37 of a maximum of 100 possible points [49]. Better primary prevention would also help reduce the risk of other non‐communicable diseases such as cardiovascular conditions. Second, EO CRC incidence in Germany remained low compared with the CRC incidence beyond 50 years of age in Germany or the EO CRC incidence in the United States. For instance, of on average 56,200 new CRC cases (including the appendix) per year in Germany between 2021 and 2023, only 3000 cases (5.4%) were EO CRC [21]. Third, there seem to be alternatives and a better use of resources. A study on the uptake of CRC screening among those 40–54 years old in Germany, for instance, concluded that in the group with a first‐degree relative with CRC, a large proportion was not in compliance with the medical guidelines recommending screening by the age of 40 at the latest [50]. Thus, the potential of a risk‐based screening has not been fully exploited. Starting population‐based screening earlier would also draw away resources, which are needed for groups with a known substantially higher CRC risk like familial predisposition, other preconditions related to CRC and older age [51].

Ongoing and future studies should aim to further elucidate the role of early‐life exposures including factors such as obesity, antibiotic use, low physical activity and dietary patterns including the consumption of ultra‐processed food, and their impact on the gut microbiota [6]. Other studies may also explore the additional effect of environmental and lifestyle factors on CRC risk in patients with a hereditary syndrome [6]. Further research is also needed on the prevalence trends of specific EO CRC risk factors over time including pathogenic germline variants and pks‐positive *Escherichia coli* to better understand their role in EO CRC increase.

A major strength of this study is that it is the first to analyse EO CRC incidence in Germany by 10‐year age groups and sex with stratification for tumour site as well as histological subtype based on large high‐quality cancer registry data. Thus, we were able to avoid confounding of EO CRC incidence trends by anatomy, that is, tumour site, and histology. We also used latest population data, which had been corrected according to the census 2022 that revealed a substantial overcount of the population, especially of young adults. Lastly, we compared incidence trends for Germany and the United States, offering two distinct views on EO CRC trends.

Study limitations include a potential selection as well as detection bias, classification changes and a potential registration bias. A selection bias might arise from selecting a subset of all German states. Our analyses, however, covered 46% of the German population. Similarly, results based on SEER 21 data may not be generalisable; however, this is the largest available SEER database covering 12 more population‐based cancer registries as compared with SEER 9, on which previous studies were based. We aimed to control for a detection bias by considering various tumour characteristics, enabling us to observe different time trends regarding histological subtype, tumour size and grading. Classification changes are an important source of bias; however, analysis of colorectum and appendix was done separately [17] and the previously borderline malignant appendix tumours affected by classification changes [29] were included in the analysis. We cannot exclude a potential registration bias due to delayed notification to the cancer registries, leading to an underestimation of CRC incidence for the most recent years. However, this bias is expected to be limited as only states with complete registration were included in the analysis.

We conclude that there has been a moderate incidence increase of EO CRC in Germany, which was limited to those aged 20–29 and 30–39 years. A large part of this increase was due to cancers with good prognosis, that is, NENs, cancers with small tumour size and cancers with low grading. However, an increase was also observed for T4 cancers in the age group 30–39 years for both males and females, which makes it likely that observed EO CRC incidence trends reflect not only earlier diagnosis but also an increased risk due to specific exposures such as obesity. In contrast to Germany, EO CRC incidence in the United States has been substantially higher throughout the years and has increased at a faster pace. Results from the United States can, therefore, not be transferred to the German population. It is important to monitor future EO CRC incidence and mortality trends. Lowering the CRC screening age in the German general population, however, does not seem warranted at present.

## Author Contributions

**Sven Voigtländer:** conceptualization, investigation, writing – original draft, data curation, formal analysis, visualization, methodology. **Hiltraud Kajüter:** conceptualization, investigation, methodology, writing – review and editing. **Ina Wellmann:** validation, writing – review and editing. **Andras Szentkirályi:** validation, writing – review and editing. **Bernd Holleczek:** writing – review and editing. **Volker Arndt:** conceptualization, writing – review and editing, supervision. **Jacqueline Müller‐Nordhorn:** conceptualization, writing – review and editing, supervision.

## Ethics Statement

The study was reviewed and approved by the Ethics Committee of the Bavarian State Chamber of Physicians, Mühlbaurstraße 16, 81677 München, Germany, Phone: +49‐(0)89‐4147‐283, Email: ethikkommission@blaek.de (reference number: 2024‐1161).

## Consent

The authors have nothing to report.

## Conflicts of Interest

The authors declare no conflicts of interest.

## Supporting information


**Table S1:** Characteristics of incident appendix cancer for ages 20–49 years in Germany, 2003–2023.
**Table S2:** Age‐standardised incidence and mortality rates of colorectal cancer for ages 20–49 in Germany, 2003 and 2023.
**Table S3:** Age‐standardised incidence rates of appendix cancer for ages 20–49 in Germany, 2003 and 2023.
**Table S4:** Average annual percent change with 95% confidence intervals for age‐standardised incidence rates of appendix cancer for ages 20–49 in Germany, 2003–2023.
**Table S5:** Annual percent change with 95% confidence intervals for age‐standardised incidence rates of colorectal cancer below age 50 in Germany, 2003–2023, and the United States, 2000–2022.
**Table S6:** Annual percent change with 95% confidence intervals for age‐standardised mortality rates of colorectal cancer below age 50 in Germany and the United States, 2000–2023.
**Figure S1:** Age‐standardised incidence rates with 95% confidence intervals of appendix cancer for ages 20–49 in Germany, 2003–2023.
**Figure S2:** Age‐standardised incidence rates with 95% confidence intervals of colorectal cancer for ages 20–49 in Germany, 2003–2023, by sex and tumour site.
**Figure S3:** Age‐standardised incidence rates with 95% confidence intervals of colorectal cancer for ages 20–49 in Germany, 2003–2023, by sex and tumour size.
**Figure S4:** Age‐standardised incidence rates with 95% confidence intervals of colorectal cancer for ages 20–49 in Germany, 2003–2023, by sex and grading.

## Data Availability

The data underlying this article (https://doi.org/10.18444/5.03.01.0005.0020.0002) can be obtained from the Centre for Cancer Registry Data (ZfKD) on reasonable request. Further information is available from the corresponding author upon request.

## References

[1] S. G. Patel , J. J. Karlitz , T. Yen , C. H. Lieu , and C. R. Boland , “The Rising Tide of Early‐Onset Colorectal Cancer: A Comprehensive Review of Epidemiology, Clinical Features, Biology, Risk Factors, Prevention, and Early Detection,” Lancet Gastroenterology & Hepatology 7 (2022): 262–274. 35090605 10.1016/S2468-1253(21)00426-X

[2] M. Araghi , I. Soerjomataram , A. Bardot , et al., “Changes in Colorectal Cancer Incidence in Seven High‐Income Countries: A Population‐Based Study,” Lancet Gastroenterology & Hepatology 4 (2019): 511–518. 31105047 10.1016/S2468-1253(19)30147-5 PMC7617144

[3] F. E. Vuik , S. A. Nieuwenburg , M. Bardou , et al., “Increasing Incidence of Colorectal Cancer in Young Adults in Europe Over the Last 25 Years,” Gut 68 (2019): 1820–1826. 31097539 10.1136/gutjnl-2018-317592 PMC6839794

[4] R. L. Siegel , S. A. Fedewa , W. F. Anderson , et al., “Colorectal Cancer Incidence Patterns in the United States, 1974‐2013,” Journal of the National Cancer Institute 109 (2017): djw322. 28376186 10.1093/jnci/djw322 PMC6059239

[5] H. Sung , R. L. Siegel , M. Laversanne , et al., “Colorectal Cancer Incidence Trends in Younger Versus Older Adults: An Analysis of Population‐Based Cancer Registry Data,” Lancet Oncology 26 (2024): 51–63. 39674189 10.1016/S1470-2045(24)00600-4 PMC11695264

[6] M. C. W. Spaander , A. G. Zauber , S. Syngal , et al., “Young‐Onset Colorectal Cancer,” Nature Reviews. Disease Primers 9 (2023): 21. 10.1038/s41572-023-00432-7 PMC10589420 37105987

[7] L. F. Tanaka , S. H. Figueroa , V. Popova , S. J. Klug , and N. Buttmann‐Schweiger , “The Rising Incidence of Early‐Onset Colorectal Cancer,” Deutsches Ärzteblatt International 120 (2023): 59–64. 36471648 10.3238/arztebl.m2022.0368 PMC10080225

[8] S. Voigtländer , A. Hakimhashemi , N. Grundmann , et al., “Trends of Colorectal Cancer Incidence According to Age, Anatomic Site, and Histological Subgroup in Bavaria: A Registry‐Based Study,” Frontiers in Oncology 12 (2022): 904546. 36212427 10.3389/fonc.2022.904546 PMC9533724

[9] R. L. Siegel , N. S. Wagle , A. Cercek , R. A. Smith , and A. Jemal , “Colorectal Cancer Statistics, 2023,” CA: A Cancer Journal for Clinicians 73 (2023): 233–254. 36856579 10.3322/caac.21772

[10] H. Brenner , P. Schrotz‐King , B. Holleczek , A. Katalinic , and M. Hoffmeister , “Declining Bowel Cancer Incidence and Mortality in Germany,” Deutsches Ärzteblatt International 113 (2016): 101–106. 26940777 10.3238/arztebl.2016.0101 PMC4791563

[11] S. Voigtländer , A. Hakimhashemi , N. Grundmann , M. Meyer , and J. Müller‐Nordhorn , “Comparison of Trends in Early‐Onset Colorectal Cancer in North America and Europe,” Lancet Gastroenterology & Hepatology 7 (2022): 505–506. 10.1016/S2468-1253(22)00094-2 35550050

[12] A. C. Chambers , S. W. Dixon , P. White , A. C. Williams , M. G. Thomas , and D. E. Messenger , “Demographic Trends in the Incidence of Young‐Onset Colorectal Cancer: A Population‐Based Study,” British Journal of Surgery 107 (2020): 595–605. 32149386 10.1002/bjs.11486 PMC7155067

[13] L. Möller , I. Wellmann , A. Stang , and H. Kajüter , “The Epidemiology of Colorectal Cancer in Younger and Older Patients,” Deutsches Ärzteblatt International 120 (2023): 277–283. 36919357 10.3238/arztebl.m2023.0041 PMC10304004

[14] R. L. Siegel , K. D. Miller , A. Goding Sauer , et al., “Colorectal Cancer Statistics, 2020,” CA: A Cancer Journal for Clinicians 70 (2020): 145–164. 32133645 10.3322/caac.21601

[15] E. M. Montminy , M. Zhou , C. Long , S. Wani , S. G. Patel , and J. J. Karlitz , “Impact of Carcinoid and Appendiceal Site Inclusion on Early Onset Colon Cancer Incidence Rate Analysis in the United States,” Clinical Gastroenterology and Hepatology 21 (2023): 2972–2974. 36273798 10.1016/j.cgh.2022.10.010

[16] E. M. Montminy , M. Zhou , L. Maniscalco , et al., “Contributions of Adenocarcinoma and Carcinoid Tumors to Early‐Onset Colorectal Cancer Incidence Rates in the United States,” Annals of Internal Medicine 174 (2021): 157–166. 33315473 10.7326/M20-0068

[17] S. K. Char , J. A. Chan , and K. Ng , “Rising Incidence of Early‐Onset Colorectal Cancer: Impact of Anatomy and Histology,” Journal of the National Cancer Institute 117 (2025): 1302–1304. 40439054 10.1093/jnci/djaf094

[18] S. G. Patel , F. P. May , J. C. Anderson , et al., “Updates on Age to Start and Stop Colorectal Cancer Screening: Recommendations From the U.S. Multi‐Society Task Force on Colorectal Cancer,” Gastroenterology 162 (2022): 285–299. 34794816 10.1053/j.gastro.2021.10.007

[19] S. K. Char , C. A. O'Connor , and K. Ng , “Early‐Onset Gastrointestinal Cancers: Comprehensive Review and Future Directions,” British Journal of Surgery 112 (2025): znaf102. 40624747 10.1093/bjs/znaf102 PMC13234757

[20] C. Pleguezuelos‐Manzano , J. Puschhof , A. Rosendahl Huber , et al., “Mutational Signature in Colorectal Cancer Caused by Genotoxic Pks(+) *E. coli* ,” Nature 580 (2020): 269–273. 32106218 10.1038/s41586-020-2080-8 PMC8142898

[21] J. Hübner , S. Voigtländer , C. Schneider , M. Pflüger , B. Holleczek , and K. Kraywinkel , “Darmkrebs in Deutschland: Inzidenz, Mortalität Und Überleben 2003 Bis 2023 [Colorectal Cancer in Germany: Incidence, Mortality, and Survival From 2003 to 2023],” Die Onkologie 31 (2025): 1089–1101.

[22] Federal Joint Committee (GBA) , “Colorectal Cancer Screening Program [Programm zur Früherkennung von Darmkrebs],” 2025.

[23] Leitlinienprogramm Onkologie (DKG, DKH, AWMF) , “Kolorektales Karzinom, Langversion 3.0 [Colorectal Cancer, Long Version 3.0],” 2025.

[24] A. Waldmann , P. Borchers , and A. Katalinic , “Temporal Trends in Age‐ and Stage‐Specific Incidence of Colorectal Adenocarcinomas in Germany,” BMC Cancer 23 (2023): 1180. 38041106 10.1186/s12885-023-11660-1 PMC10693075

[25] J. B. O'Connell , M. A. Maggard , J. H. Liu , D. A. Etzioni , E. H. Livingston , and C. Y. Ko , “Rates of Colon and Rectal Cancers Are Increasing in Young Adults,” American Surgeon 69 (2003): 866–872. 14570365

[26] Zentrum für Krebsregisterdaten (ZfKD) , “Datensatz des ZfKD auf Basis der epidemiologischen Landeskrebsregisterdaten Epi2023_3, verfügbare Diagnosejahre bis 2023,” 2025.

[27] V. Arndt , B. Holleczek , H. Kajuter , et al., “Data From Population‐Based Cancer Registration for Secondary Data Analysis: Methodological Challenges and Perspectives,” Gesundheitswesen 82 (2020): S62–S71. 31663107 10.1055/a-1009-6466

[28] SEER*Explorer , An Interactive Website for SEER Cancer Statistics. Data Source(s): SEER Incidence Data, November 2024 Submission (1975–2022), SEER 21 Registries: Surveillance Research Program (National Cancer Institute, 2025).

[29] A. Fritz , C. Percy , A. Jack , et al., eds., International Classification of Diseases for Oncology, Third ed. (First Revision, World Health Organization, 2013).

[30] J. D. Brierley , M. K. Gospodarowicz , and C. Wittekind , eds., TNM Classification of Malignant Tumours, 8th ed. (Union for International Cancer Control, 2017).

[31] R. Doll and P. Cook , “Summarizing Indices for Comparison of Cancer Incidence Data,” International Journal of Cancer 2 (1967): 269–279. 6041994 10.1002/ijc.2910020310

[32] R. C. Tiwari , L. X. Clegg , and Z. Zou , “Efficient Interval Estimation for Age‐Adjusted Cancer Rates,” Statistical Methods in Medical Research 15 (2006): 547–569. 17260923 10.1177/0962280206070621

[33] H. J. Kim , H. S. Chen , J. Byrne , B. Wheeler , and E. J. Feuer , “Twenty Years Since Joinpoint 1.0: Two Major Enhancements, Their Justification, and Impact,” Statistics in Medicine 41 (2022): 3102–3130. 35522060 10.1002/sim.9407

[34] R. N. Anderson and H. M. Rosenberg , “Age Standardization of Death Rates: Implementation of the Year 2000 Standard,” National Vital Statistics Reports 47 (1998): 1–16. 9796247

[35] Surveillance Research Program, National Cancer Institute , “Joinpoint Regression Software, Version 5.4.0,” 2025.

[36] L. Möller , A. Szentkirályi , C. Eisfeld , et al., “Incidence Trends and Relative Survival of Colorectal Neuroendocrine Neoplasms: A Population‐Based Study Using German Cancer Registry Data,” International Journal of Cancer 157 (2025): 116–125. 39976321 10.1002/ijc.35372 PMC12062926

[37] H. Li , D. Boakye , X. Chen , M. Hoffmeister , and H. Brenner , “Association of Body Mass Index With Risk of Early‐Onset Colorectal Cancer: Systematic Review and Meta‐Analysis,” American Journal of Gastroenterology 116 (2021): 2173–2183. 34309586 10.14309/ajg.0000000000001393 PMC8560162

[38] H. Li , D. Boakye , X. Chen , et al., “Associations of Body Mass Index at Different Ages With Early‐Onset Colorectal Cancer,” Gastroenterology 162 (2021): 1088–1097.e3. 34914944 10.1053/j.gastro.2021.12.239

[39] G. B. Mensink , A. Schienkiewitz , M. Haftenberger , T. Lampert , T. Ziese , and C. Scheidt‐Nave , “Übergewicht und Adipositas in Deutschland: Ergebnisse der Studie zur Gesundheit Erwachsener in Deutschland (DEGS1) [Overweight and obesity in Germany: results of the German Health Interview and Examination Survey for Adults (DEGS1)],” Bundesgesundheitsblatt, Gesundheitsforschung, Gesundheitsschutz 56 (2013): 786–794. 23703499 10.1007/s00103-012-1656-3

[40] A. Schienkiewitz , A.‐K. Brettschneider , S. Damerow , and R. A. Schaffrath , “Übergewicht und Adipositas im Kindes‐ und Jugendalter in Deutschland – Querschnittergebnisse aus KiGGS Welle 2 und Trends,” Journal of Health Monitoring 3 (2018): 16–23.

[41] C. M. Hales , C. D. Fryar , M. D. Carroll , D. S. Freedman , and C. L. Ogden , “Trends in Obesity and Severe Obesity Prevalence in US Youth and Adults by Sex and Age, 2007‐2008 to 2015‐2016,” JAMA 319 (2018): 1723–1725. 29570750 10.1001/jama.2018.3060 PMC5876828

[42] M. Hornschuch , S. Schwarz , and U. Haug , “10‐Year Prevalence of Diagnostic and Screening Colonoscopy Use in Germany: A Claims Data Analysis,” European Journal of Cancer Prevention 31 (2022): 497–504. 34983895 10.1097/CEJ.0000000000000736 PMC9928559

[43] H. Tillmanns , G. Schillinger , and H. Dräther , “Inanspruchnahme von Früherkennungsleistungen der gesetzlichen Krankenversicherung durch AOK‐Versicherte im Erwachsenenalter (2007 bis 2021) [Use of Early Detection Services Provided of the Statutory Health Insurance by AOK Insured Persons of Adult Age (2007‐2021)],” 2022, Wissenschaftliches Institut der AOK (WIdO).

[44] S. Voigtländer , A. Hakimhashemi , N. Grundmann , et al., “Impact of the COVID‐19 Pandemic on Reported Cancer Diagnoses in Bavaria, Germany,” Journal of Cancer Research and Clinical Oncology 149 (2023): 7493–7503. 36964405 10.1007/s00432-023-04707-0 PMC10038367

[45] US Preventive Services Task Force , K. W. Davidson , M. J. Barry , et al., “Screening for Colorectal Cancer: US Preventive Services Task Force Recommendation Statement,” JAMA 325 (2021): 1965–1977.34003218

[46] Institut für Qualität und Wirtschaftlichkeit im Gesundheitswesen (IQWiG) , “Überprüfung der unteren Altersgrenze und Frequenz der Früherkennungskoloskopie im Darmkrebs‐Screeningprogramm, Abschlussbericht [Review of the Lower Age Limit and Frequency of Screening Colonoscopy in the Colorectal Cancer Screening Program: Final Report],” 2026.

[47] J. M. G. Wilson and G. Jungner , Principles and Practice of Screening for Disease (World Health Organization, 1968).

[48] WHO Regional Office for Europe , “A Short Guide to Cancer Screening: Increase Effectiveness, Maximize Benefits and Minimize Harm,” 2022.

[49] AOK‐Bundesverband and Deutsches Krebsforschungszentrum (DKFZ) , “Public Health Index: Gesundheitssschutz im Europäischen Vergleich [Public Health Index: Health Protection in European Comparison],” 2025.

[50] K. Weigl , K. Tikk , M. Hoffmeister , et al., “Prevalence of a First‐Degree Relative With Colorectal Cancer and Uptake of Screening Among Persons 40 to 54 Years Old,” Clinical Gastroenterology and Hepatology 18 (2020): 2535–2543. 31809916 10.1016/j.cgh.2019.11.044

[51] M. Hoffmeister and H. Brenner , “Darmkrebs Bei Jungen Erwachsenen [Colorectal Cancer in Young Adults],” Die Gastroenterologie 19 (2024): 396–403.

