# Supplementary material for: Incidence Trends of Early‐Onset Colorectal Cancer in Germany: A Registry‐Based Study From 2003 to 2023
Source: Int J Cancer. 2026 Jun 23;159(9):2207–18. doi: 10.1002/ijc.70600 (PMC13548003; doi:10.1002/ijc.70600)
Supplement: Supplementary file 1 [file IJC-159-2207-s001.pdf]

SUPPLEMENT

Incidence trends of early-onset colorectal cancer in Germany: A registry-based study from 2003 to 2023

Sven Voigtländer, Hiltraud Kajüter, Ina Wellmann, Andras Szentkirályi, Bernd Holleczek, Volker Arndt, Jacqueline Müller-Nordhorn and the ALSTER Working Group

Further members of the ALSTER (Altersspezifische Trends bei den Krebsneuerkrankungen [Age-specific trends in cancer incidence]) Working Group:
Josephine Kanbach, Sabine Luttmann, Meike Ressing, Cécile Ronckers, Soo-Zin Kim-Wanner, Alice Nennecke, Frederik Peters, Klaus Kraywinkel, Roxana Müller-Eberstein, Hannah Baltus, Paula Grieger, Antje Schliemann, Annika Waldmann

Table of contents

1. Supplementary Tables 2
2. Supplementary Figures 8

1. Supplementary Tables

Supplementary Table 1: Characteristics of incident appendix cancer for ages 20-49 years in Germany, 2003-2023.

	20-29 years				30-39 years				40-49 years				Total			
	Male		Female		Male		Female		Male		Female		Male		Female	
	n	%	n	%	n	%	n	%	n	%	n	%	n	%	n	%
Total	306	100	556	100	369	100	531	100	560	100	647	100	1,235	100	1,734	100
Histological subtype																
Adenocarcinoma	28	9.2	29	5.2	91	24.7	81	15.3	224	40.0	209	32.3	343	27.8	319	18.4
Neuroendocrine neoplasm	273	89.2	517	93.0	242	65.6	418	78.7	243	43.4	378	58.4	758	61.4	1,313	75.7
Other specific	3	1.0	5	0.9	28	7.6	21	4.0	75	13.4	48	7.4	106	8.6	74	4.3
Unspecified	2	0.7	5	0.9	8	2.2	11	2.1	18	3.2	12	1.9	28	2.3	28	1.6
Tumour size																
T1	142	46.4	246	44.2	118	32.0	207	39.0	146	26.1	161	24.9	406	32.9	614	35.4
T2	18	5.9	41	7.4	26	7.0	41	7.7	34	6.1	37	5.7	78	6.3	119	6.9
T3	74	24.2	98	17.6	80	21.7	108	20.3	146	26.1	135	20.9	300	24.3	341	19.7
T4	22	7.2	40	7.2	54	14.6	65	12.2	137	24.5	158	24.4	213	17.2	263	15.2
Unknown	50	16.3	131	23.6	91	24.7	110	20.7	97	17.3	156	24.1	238	19.3	397	22.9
Grading																
Low	230	75.2	397	71.4	200	54.2	359	67.6	237	42.3	303	46.8	667	54.0	1,059	61.2
Intermediate	25	8.2	37	6.7	53	14.4	44	8.3	107	19.1	79	12.2	185	15.0	160	9.2
High	9	2.9	4	0.7	33	8.9	28	5.3	97	17.3	92	14.2	139	11.3	124	7.2
Unknown	42	13.7	118	21.2	83	22.5	100	18.8	119	21.3	173	26.7	244	19.8	391	22.5

Notes: T, tumour size according to the TNM Classification of Malignant Tumours (8th edition). ICD-10 codes C18.1.

Supplementary Table 2: Age-standardised incidence and mortality rates of colorectal cancer for ages 20-49 in Germany, 2003 and 2023.

	20-29 years				30-39 years				40-49 years			
	Male		Female		Male		Female		Male		Female	
Incidence	2003	2023	2003	2023	2003	2023	2003	2023	2003	2023	2003	2023
Total	0.9	1.7	0.8	1.9	4.3	6.3	3.7	5.2	18.1	20.7	17.0	18.0
Tumour site												
Proximal colon	0.2	0.5	0.1	0.4	1.2	1.6	1.0	1.0	3.6	4.8	3.1	3.6
Distal colon	0.3	0.5	0.3	0.5	1.2	1.6	0.8	1.9	4.2	5.7	5.0	5.9
Rectum	0.2	0.5	0.2	0.8	1.3	2.8	1.5	2.0	9.1	9.4	7.1	7.6
Overlapping lesion	0.0	<0.1	<0.1	0.1	<0.1	<0.1	0.1	0.0	0.1	0.3	0.1	0.2
Unspecified	0.2	0.1	<0.1	<0.1	0.6	0.2	0.3	0.2	1.1	0.6	1.7	0.6
Histological subtype												
Adenocarcinoma	0.7	1.2	0.5	1.4	4.0	5.3	3.4	4.4	16.3	19.2	15.2	16.2
Neuroendocrine neoplasm	0.0	0.2	0.0	0.3	0.2	0.7	<0.1	0.5	0.2	1.0	0.3	1.2
Other specific	0.0	0.1	0.0	0.0	0.0	0.1	<0.1	<0.1	0.1	<0.1	0.2	0.1
Unspecified	0.2	0.2	0.2	0.1	0.2	0.3	0.2	0.2	1.5	0.5	1.3	0.5
Tumour size												
T1	0.0	0.2	0.0	0.2	0.2	1.0	0.3	0.8	1.2	2.8	1.5	2.5
T2	0.1	0.3	0.0	0.2	0.4	0.6	0.2	0.4	2.5	1.8	2.3	1.7
T3	0.5	0.7	0.3	0.4	1.9	2.3	1.7	2.2	8.2	8.2	7.5	6.8
T4	<0.1	0.3	0.1	0.3	0.9	0.8	0.5	0.9	2.6	3.1	2.3	3.0
Unknown	0.2	0.2	0.3	0.7	0.9	1.6	0.9	0.9	3.6	4.8	3.4	4.1
Grading												
Low	0.0	0.5	0.0	0.3	0.2	1.5	0.2	1.1	0.9	4.1	0.8	3.4
Intermediate	0.5	0.6	0.2	0.8	2.1	2.8	2.0	2.5	11.3	11.2	10.2	10.4
High	0.2	0.2	0.1	0.4	1.4	1.3	1.1	0.9	3.7	3.3	3.7	2.5
Unknown	0.2	0.4	0.4	0.3	0.6	0.8	0.4	0.6	2.2	2.1	2.3	1.7
	20-29 years				30-39 years				40-49 years			
	Male		Female		Male		Female		Male		Female	
Mortality	2003	2023	2003	2023	2003	2023	2003	2023	2003	2023	2003	2023
Total[1]	0.3	0.4	0.1	0.3	1.2	1.7	0.8	0.9	6.1	5.6	4.8	3.6

Notes: T, tumour size according to the TNM Classification of Malignant Tumours (8th edition). Age-standardised incidence rates per 100,000 based on the old European standard population. ICD-10 codes C18-C20 excluding C18.1. [1] Mortality due to ICD-10 codes C18-C20 including C18.1.

Supplementary Table 3: Age-standardised incidence rates of appendix cancer for ages 20-49 in Germany, 2003 and 2023.

	20-29 years				30-39 years				40-49 years			
	Male		Female		Male		Female		Male		Female	
	2003	2023	2003	2023	2003	2023	2003	2023	2003	2023	2003	2023
Total	0.1	0.9	0.5	1.6	0.3	1.1	0.4	1.1	0.4	1.1	0.8	1.2
Histological subtype												
Adenocarcinoma	0.1	0.0	0.0	0.2	<0.1	0.3	0.1	<0.1	0.2	0.4	0.2	0.3
Neuroendocrine neoplasm	<0.1	0.9	0.5	1.4	0.3	0.7	0.3	0.9	0.1	0.5	0.5	0.8
Other specific	0.0	0.0	0.0	0.0	0.1	0.1	0.0	0.1	<0.1	0.1	<0.1	0.1
Unspecified	0.0	0.0	0.0	0.0	0.0	0.0	0.0	<0.1	0.0	<0.1	<0.1	0.0
Tumour size												
T1	0.0	0.3	0.0	0.7	<0.1	0.3	0.0	0.5	0.1	0.3	0.0	0.2
T2	0.0	0.1	0.0	0.2	0.0	<0.1	0.0	<0.1	0.1	0.1	0.1	0.1
T3	0.1	0.4	0.1	0.6	0.1	0.4	0.1	0.4	0.1	0.3	0.3	0.5
T4	0.1	0.0	0.0	0.2	<0.1	0.3	<0.1	0.1	0.1	0.4	0.1	0.3
Unknown	<0.1	0.1	0.3	<0.1	0.2	0.1	0.3	<0.1	0.1	0.1	0.4	0.1
Grading												
Low	<0.1	0.7	0.2	1.3	0.1	0.7	0.1	0.9	0.1	0.5	0.2	1.0
Intermediate	<0.1	<0.1	0.0	0.2	<0.1	0.2	0.0	0.1	0.1	0.3	0.2	<0.1
High	0.0	<0.1	0.0	0.0	<0.1	0.1	<0.1	<0.1	<0.1	0.1	0.1	<0.1
Unknown	<0.1	<0.1	0.2	0.2	0.2	<0.1	0.2	<0.1	0.1	0.2	0.3	0.1

Notes: T, tumour size according to the TNM Classification of Malignant Tumours (8th edition). Age-standardised incidence rates per 100,000 based on the old European standard population. ICD-10 codes C18.1.

Supplementary Table 4: Average annual percent change with 95% confidence intervals for age-standardised incidence rates of appendix cancer for ages 20-49 in Germany, 2003-2023.

	20-29 years				30-39 years				40-49 years			
	Male		Female		Male		Female		Male		Female	
	AAPC	95%-CI	AAPC	95%-CI	AAPC	95%-CI	AAPC	95%-CI	AAPC	95%-CI	AAPC	95%-CI
Total	6.8	[3.8; 13.5]	5.3	[1.2; 12.5]	5.4	[3.8; 7.5]	6.4	[4.0; 9.9]	4.0	[0.9; 10.2]	3.3	[1.5; 5.2]
Histological subtype												
Adenocarcinoma	NA		NA		4.1	[1.5; 7.5]	5.4	[1.2; 11.5]	7.2	[2.9; 13.8]	3.8	[-0.1; 8.1]
Neuroendocrine neoplasm	7.9	[4.2; 18.3]	5.8	[2.1; 11.6]	5.6	[3.7; 8.1]	6.9	[4.8; 10.1]	5.8	[2.4; 10.4]	2.1	[-2.7; 8.5]
Other specific	NA		NA		NA		NA		4.1	[-0.0; 9.2]	4.1	[0.9; 8.0]
Unspecified	NA		NA		NA		NA		NA		NA	
Tumour size												
T1	NA		NA		NA		NA		12.3	[7.4;25.4]	NA	
T2	NA		NA		NA		NA		NA		NA	
T3	NA		NA		NA		NA		6.5	[3.3; 10.7]	4.1	[0.8; 9.6]
T4	NA		NA		NA		NA		9.0	[5.6; 14.3]	5.1	[0.3; 11.1]
Unknown	NA		NA		-5.9	[-10.4; -3.1]	-7.0	[-19.1; -2.5]	-0.3	[-4.5; 3.4]	-7.8	[-17.0; -5.7]
Grading												
Low	13.3	[10.4; 21.9]	8.6	[5.2; 15.5]	9.5	[7.2; 13.5]	12.2	[9.4; 18.7]	9.7	[5.3; 17.4]	7.7	[2.3; 20.1]
Intermediate	NA		NA		NA		NA		NA		2.6	[-1.0; 6.5]
High	NA		NA		NA		NA		2.8	[-0.7; 6.7]	2.1	[-11.0; 14.0]
Unknown	NA		NA		-4.7	[-8.5; -1.9]	-7.8	[-19.4; -1.0]	0.4	[-3.8; 4.1]	-3.4	[-6.9; -0.9]

Notes: AAPC, average annual percent change; CI, confidence interval; T, tumour size according to the TNM Classification of Malignant Tumours (8th edition); NA, not applicable due to years with zero cases. Age-standardised rates per 100,000 based on the old European standard population. ICD-10 codes C18.1.

Supplementary Table 5: Annual percent change with 95% confidence intervals for age-standardised incidence rates of colorectal cancer below age 50 in Germany, 2003-2023, and the United States, 2000-2022.

	Germany			United States		
	Period	APC[1]	95%-CI	Period	APC[1]	95%-CI
Males	2003-2023	0.8	[0.3; 1.4]	2000-2016	1.2	[0.8; 1.4]
				2016-2022	3.9	[2.9; 6.0]
Females	2003-2023	1.0	[0.6; 1.4]	2000-2019	1.1	[0.8; 1.4]
				2019-2022	7.0	[4.0; 8.4]

Notes: APC, annual percent change; CI, confidence interval. Age-standardised rates per 100,000 based on the 2000 US standard population. ICD-10 codes C18-C20 excluding C18.1. Germany: Analysis set; United States: SEER 21 registries. [1] The year 2020 was excluded from the model fitting.

Supplementary Table 6: Annual percent change with 95% confidence intervals for age-standardised mortality rates of colorectal cancer below age 50 in Germany and the United States, 2000-2023.

	Germany			United States		
	Period	APC	95%-CI	Period	APC	95%-CI
Males	2000-2014	-1.7	[-3.3; 1.1]	2000-2004	-2.1	[-5.9; 0.5]
	2014-2023	1.3	[-0.1; 6.1]	2004-2023	1.3	[1.0; 2.0]
Females	2000-2006	-3.7	[-10.2; -1.0]	2000-2004	-1.1	[-4.2; 0.8]
	2006-2023	0.1	[-0.6; 2.8]	2004-2023	0.8	[-0.1; 2.4]

Notes: APC, annual percent change; CI, confidence interval. Age-standardised rates per 100,000 based on the 2000 US standard population. ICD-10 codes C18-C20 including C18.1. Germany: All states; United States: All states.

2. Supplementary Figures

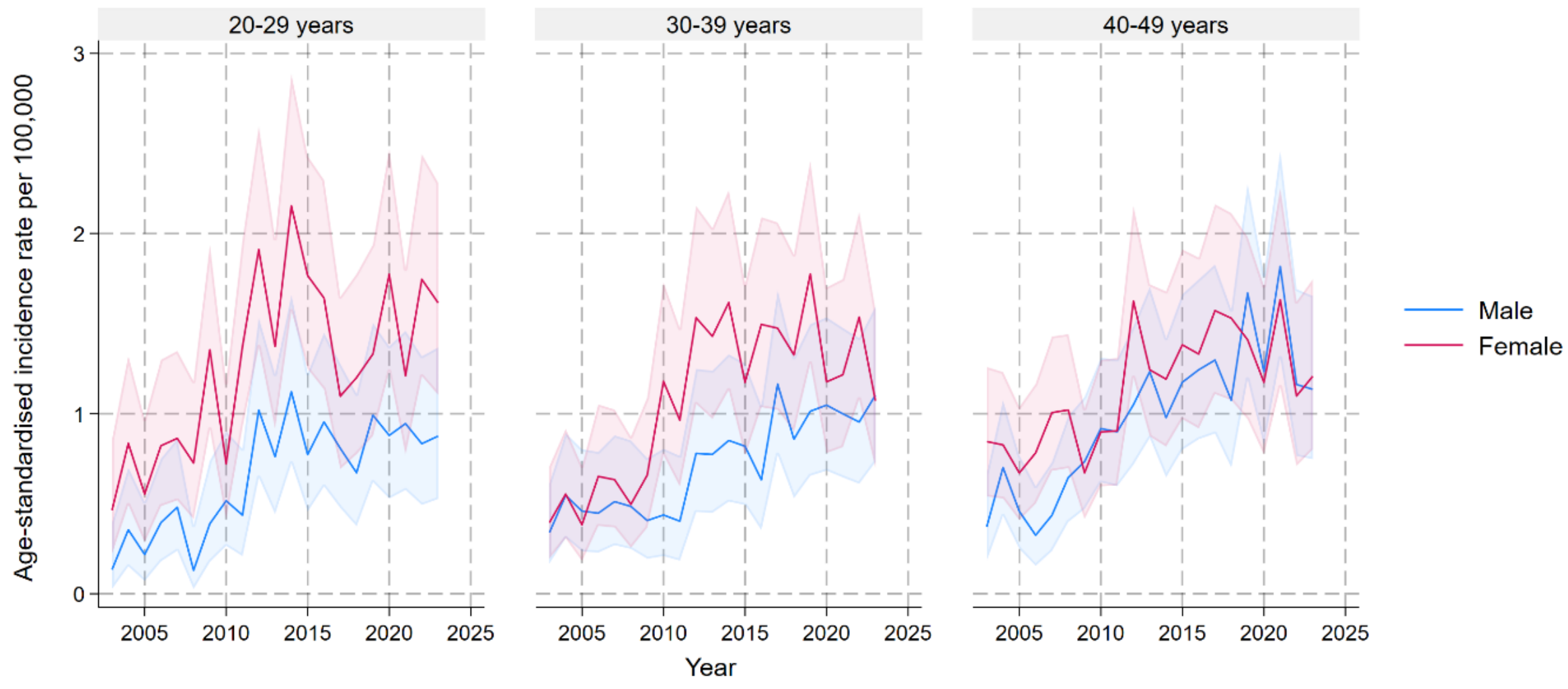

Supplementary Figure 1: Age-standardised incidence rates with 95% confidence intervals of appendix cancer for ages 20-49 in Germany, 2003-2023.

Notes: Age-standardised incidence rates per 100,000 based on the old European standard population. ICD-10 codes C18.1.

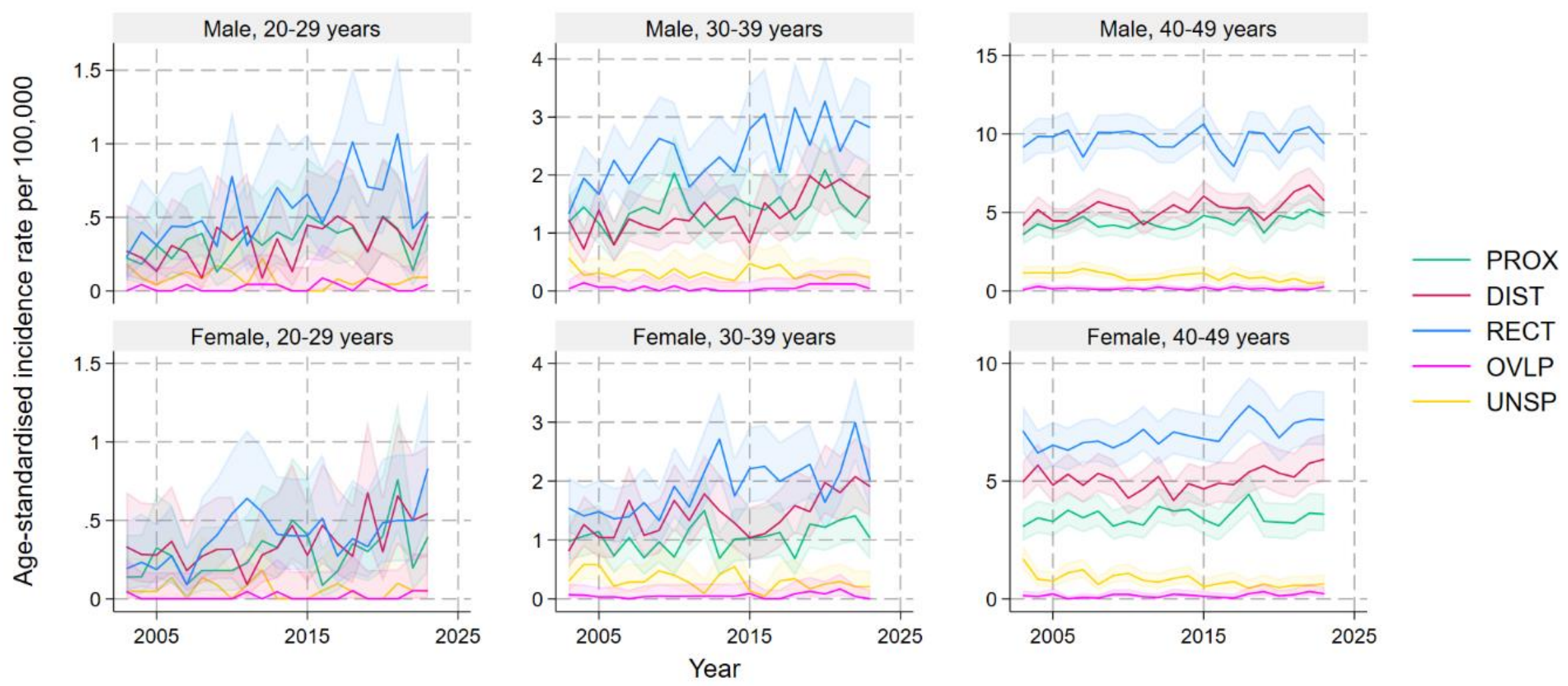

Supplementary Figure 2: Age-standardised incidence rates with 95% confidence intervals of colorectal cancer for ages 20-49 in Germany, 2003-2023, by sex and tumour site.

Notes: ICD-10 codes C18-C20 excluding C18.1. PROX, proximal colon; DIST, distal colon; RECT, rectum; OVLP, overlapping lesion; UNSP, unspecified cancers. Age-standardised incidence rates per 100,000 based on the old European standard population. Scale of y-axis varies.

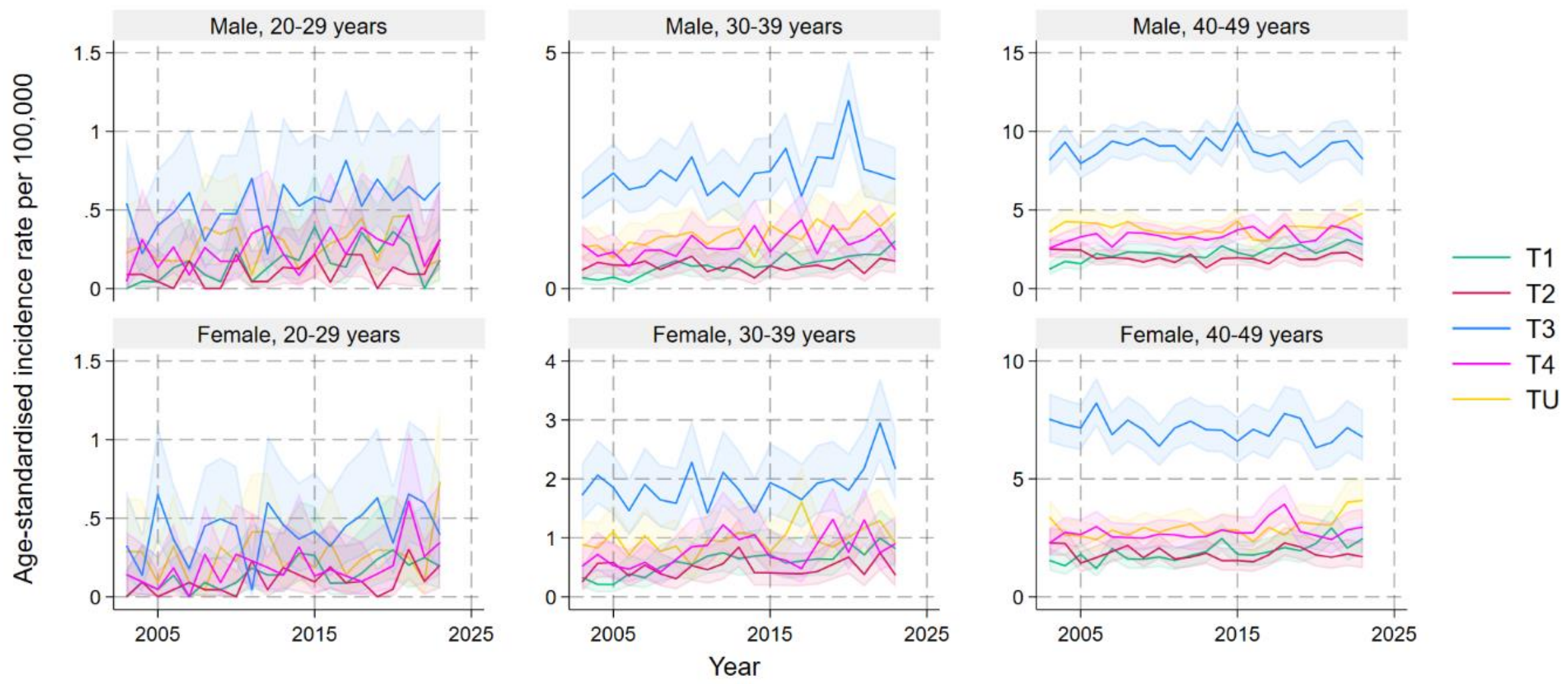

Supplementary Figure 3: Age-standardised incidence rates with 95% confidence intervals of colorectal cancer for ages 20-49 in Germany, 2003-2023, by sex and tumour size.

Notes: ICD-10 codes C18-C20 excluding C18.1. T, tumour size according to the TNM Classification of Malignant Tumours (8th edition); TU, tumour size unknown. Age-standardised incidence rates per 100,000 based on the old European standard population. Scale of y-axis varies.

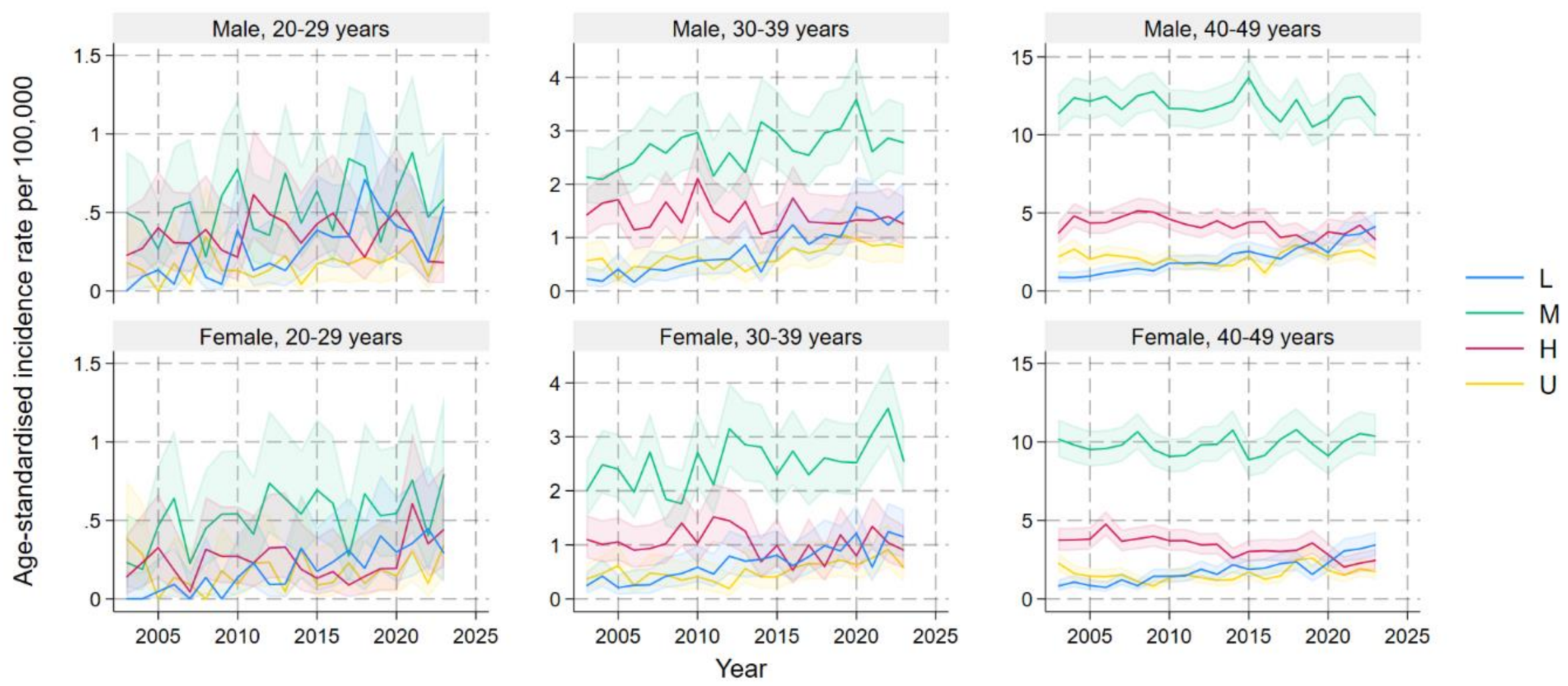

Supplementary Figure 4: Age-standardised incidence rates with 95% confidence intervals of colorectal cancer for ages 20-49 in Germany, 2003-2023, by sex and grading.

Notes: ICD-10 codes C18-C20 excluding C18.1. L, low grading; M, intermediate grading; H, high grading; U, unknown grading. Age-standardised incidence rates per 100,000 based on the old European standard population. Scale of y-axis varies.
